# Socioeconomic Factors Associated With Diet Quality in Pregnancy: A Cross‐Sectional Australian Study

**DOI:** 10.1111/mcn.70170

**Published:** 2026-02-12

**Authors:** Bree Whiteoak, Danielle Gallegos, Severine Navarro, Leonie Callaway, Susan de Jersey, Victoria Eley, Alka Kothari, Samantha L. Dawson

**Affiliations:** ^1^ School of Exercise and Nutrition Sciences Queensland University of Technology (QUT) Kelvin Grove Queensland Australia; ^2^ Centre for Childhood Nutrition Research, Faculty of Health Queensland University of Technology (QUT) South Brisbane Queensland Australia; ^3^ QIMR Berghofer Medical Research Institute Herston Queensland Australia; ^4^ Faculty of Medicine The University of Queensland Herston Queensland Australia; ^5^ Women's and Newborns Services Royal Brisbane and Women's Hospital Herston Queensland Australia; ^6^ Department of Dietetics and Foodservices Royal Brisbane and Women's Hospital Herston Queensland Australia; ^7^ Centre for Health Services Research, Faculty of Medicine The University of Queensland Herston Queensland Australia; ^8^ Department of Anaesthesia and Perioperative Medicine Royal Brisbane and Women's Hospital Herston Queensland Australia; ^9^ Redcliffe Hospital Redcliffe Queensland Australia; ^10^ IMPACT – the Institute for Mental and Physical Health and Clinical Translation, Food & Mood Centre, School of Medicine, Barwon Health Deakin University Geelong Victoria Australia

**Keywords:** diet, dietary patterns, low socioeconomic status, pregnancy, social class, socioeconomic disparities in health, socioeconomic factors

## Abstract

Prenatal diet affects maternal and child health; however, adherence to dietary guidelines in pregnancy is low. This cross‐sectional study aimed to describe overall diet quality and to examine relationships between socioeconomic factors and diet quality in a sample of Australian pregnant women. Participants (*n* = 1580) completed an online survey and self‐reported usual dietary intake (via a food frequency questionnaire [FFQ]) and socioeconomic factors, including highest educational attainment, income, perception of overall financial situation, residential postcode for area‐level socioeconomic status (SES), stressful life events, and perceived social support. FFQ responses were converted to an overall diet quality score using the Dietary Guidelines Index 2013 (DGI‐13) criteria. Latent class analysis was used to identify groups of stressful life events, and multiple linear regression models examined associations between the socioeconomic factors and DGI‐13 score. Overall, adherence to dietary guidelines and prenatal diet quality were low. The mean DGI‐13 score was 76.1 (SD 13.7) out of a maximum possible score of 130. All socioeconomic factors were significantly associated with DGI‐13 score. For all socioeconomic factors except the perceived social support score, the lowest/most disadvantaged categories and middle/medium categories were associated with clinically important reductions of 5–9 points and 3–6 points, respectively, indicating a social gradient in diet quality. There is a need to improve prenatal diet quality among all women. However, there is an urgent need for systems‐level interventions and policy change that target those with lower SES backgrounds to reduce dietary and health inequities.

## Summary

- In this sample, prenatal diet quality was generally poor.
- Various socioeconomic factors, including education, income adjusted for household composition and size, perception of financial situation, area‐level socioeconomic status, perceived social support, and latent classes of stressful life events were significantly associated with prenatal diet quality.
- Perceived social support score was not related to a clinically important change in diet quality. For all other socioeconomic factors, lower/more disadvantaged categories were associated with clinically relevant decreases in diet quality.
- While there is a need to improve diet quality among all pregnant women, systems‐level interventions and policy change targeting those with lower socioeconomic status are urgently required to reduce dietary inequities.

## 1 Introduction

Maternal diet in pregnancy can have profound effects on maternal and child health (Abdollahi et al. 2021; Davies et al. 2016). Greater adherence to a healthy prenatal diet has been associated with reduced risk of numerous adverse perinatal outcomes, including hypertensive disorders of pregnancy, maternal depression, preterm birth, and low birth weight (Abdollahi et al. 2021; Xu et al. 2024). It has also been related to cognitive and behavioural outcomes in children (Borge et al. 2017; Polanska et al. 2021; Zupo et al. 2024), highlighting the importance of optimising dietary intake in this critical period. However, studies consistently report low adherence to dietary guidelines among pregnant women in Australia and other high‐income countries (Rahmannia et al. 2024), indicating that prenatal diet quality is poor overall. Diet quality indices are commonly used to measure adherence to country‐specific dietary guidelines (Hlaing‐Hlaing et al. 2020). For example, the Dietary Guidelines Index 2013 (DGI‐13) (Thorpe et al. 2016) measures adherence to the Australian Dietary Guidelines (National Health and Medical Research Council 2013), with higher DGI‐13 scores reflecting higher overall diet quality (Thorpe et al. 2016). Understanding what factors may be precluding women from consuming a high‐quality diet in pregnancy is important to inform future interventions and public health messaging.

Health behaviours, such as dietary practices, are not simply an expression of individual choice (Locke 2022). Social factors shape behaviour (Alderwick and Gottlieb 2019); thus, dietary ‘choices’ are made within a broader socio‐cultural context and are constrained by socioeconomic factors and structural inequalities (Locke 2022). Education and income are the most common socioeconomic factors studied in dietary research, either in their own right or as proxies for socioeconomic status (SES) (Aubert et al. 2022; Doyle et al. 2017; Lewis and Lee 2021; Rahmannia et al. 2024). Higher education and income have been associated with higher prenatal diet quality in the United States (US), United Kingdom (UK), Canada, and some European countries (Aubert et al. 2022; Doyle et al. 2017; Yu et al. 2022). However, research on socioeconomic factors and prenatal diet quality in Australia is limited. Two recent pilot studies found no association between education and prenatal diet quality score (Maneschi et al. 2023) or meeting food group recommendations (Wilkinson et al. 2022), or between income and meeting food group recommendations in pregnancy (Wilkinson et al. 2022). Findings from a recent scoping review (Lewis and Lee 2021) indicate that diet is generally poorer in lower socioeconomic groups compared to higher socioeconomic groups (most commonly defined as higher education, income, and/or area‐level SES) in the broader Australian population. However, most of the dietary data included in this review were collected over a decade ago, prior to the COVID‐19 pandemic and the current cost‐of‐living crisis (Australian Bureau of Statistics 2025), and very few studies measured overall diet quality.

Socioeconomic factors beyond education and income (and, to a lesser extent, employment/occupation) are underexplored in prenatal dietary research (Aubert et al. 2022; Doyle et al. 2017; Rahmannia et al. 2024). Other factors, such as low social support and stressful life events, many of which may be considered social risk factors (e.g., exposure to violence and homelessness) (Alderwick and Gottlieb 2019), may impact a significant number of pregnant women. Around two‐thirds of women in Australia experienced at least one stressful life event in the 12 months prior to giving birth (Brown et al. 2011), similar to the US (Mukherjee et al. 2017). Additionally, a low level of social support has been reported in 7.1% of Australian pregnant women (Bedaso et al. 2021). These social factors may influence diet through multiple pathways. For example, they may affect the household's financial situation (Al‐Mutawtah et al. 2023; Bedaso et al. 2021; Brown et al. 2011; Mukherjee et al. 2017), thereby impacting ability to afford enough nutritious food to meet dietary needs and preferences (Lewis and Lee 2021; Temple 2018). They also contribute to higher psychosocial stress (Bedaso et al. 2021; Kingston et al. 2012), which influences eating behaviours (Hill et al. 2022) and has been associated with poorer diet quality among pregnant women (Fowles et al. 2012) and women of reproductive age (Khaled et al. 2020).

More research is needed to better understand the relationship between a range of social and economic indicators and overall diet quality in pregnancy, particularly in the Australian context. Therefore, this study aimed to (1) describe diet quality in a sample of Australian pregnant women, and (2) to examine associations between socioeconomic factors and overall prenatal diet quality score.

## 2 Methods

### 2.1 Study Design and Recruitment

A cross‐sectional online survey hosted on Qualtrics was conducted from August 2022 to March 2023, with an estimated completion time of 25–35 min. Eligibility criteria included being pregnant (any gestation), aged ≥ 16 years, and English proficiency. Gender was not assessed; however, for clarity (Gribble et al. 2022), participants will be referred to as pregnant women as recruitment materials and methods targeted women and mothers. The authors acknowledge that not all people who are pregnant identify as women or mothers and affirm that all care should be respectful and responsive to individual needs and preferences.

Participants were recruited via flyers displayed and/or distributed in antenatal clinics at three public hospitals in South‐East Queensland, Australia, and via paid (Meta) and unpaid (Facebook group posts) social media advertising. These advertisements targeted pregnant women residing in South‐East Queensland, however recruitment was not limited to this region due to snowball sampling from user interactions with social media posts. As people living with disadvantage are often underrepresented in research (Gallegos et al. 2023), lower SES areas, defined by lower Socio‐Economic Indexes for Areas (SEIFA) scores (Australian Bureau of Statistics 2023c), were oversampled to attempt to recruit pregnant women from a range of socioeconomic backgrounds. As a gesture of appreciation, participants were offered entry in a prize draw to win one of three AUD $200 gift cards.

Ethics approval for this study was granted by the Metro North Human Research Ethics Committee A (reference: HREC/2022/QRBW/82273) and ratified by the Queensland University of Technology (QUT) and QIMR Berghofer Medical Research Institute Human Research Ethics Committees. All participants provided informed consent online before accessing the survey.

This study is reported in accordance with the STROBE checklist for cross‐sectional studies (von Elm et al. 2007) and the Checklist for Reporting Results of Internet E‐Surveys (Eysenbach 2004) (Tables S1 and S2 in Supporting Information files S10 and S11).

### 2.2 Dietary Intake Assessment

A 107‐item semi‐quantitative food frequency questionnaire (FFQ) was used to assess dietary intake over the past 6 months, and short dietary questions assessed usual fruit and vegetable intake, salt use, trimming of fat from meat, and usual type of bread and milk consumed (Coyne et al. 2005; McLennan and Podger 1998; Rutishauser et al. 2001). Currently, there are no Australian FFQs that have been developed and validated specifically for pregnant women (Bezerra et al. 2023). The FFQ used in this study was based on a modified version of the 1995 National Nutrition Survey (McLennan and Podger 1998) that has been used previously with Australian adults of reproductive age (Smith et al. 2010). It was further modified for this study by removing the alcohol items (replaced with a single question assessing frequency of alcohol intake in the current pregnancy) and adding three extra food items (muesli bars and other snack bars; fried chicken; and Chinese/Thai/Indian takeaway) that are commonly included in other Australian FFQs and studies (Collins et al. 2014; Smith et al. 2010). FFQ responses were on a 9‐point scale, ranging from ‘never or less than once per month’ to ‘6+ times per day’. Daily equivalent servings were calculated for each item, with one serving assumed for each eating occasion as per previous applications of this FFQ (McNaughton et al. 2008; Smith et al. 2010; Thorpe et al. 2016).

### 2.3 Diet Quality Score

FFQ and short dietary question responses were converted into diet quality scores using DGI‐13 criteria (Thorpe et al. 2016), modified for pregnancy as described previously (Whiteoak et al. 2024). Higher scores reflect greater adherence to the Australian Dietary Guidelines (National Health and Medical Research Council 2013), which are recommended in the current Australian clinical practice guidelines for pregnancy care (National Health and Medical Research Council 2020). There are no Australian diet quality indices that have been validated specifically for pregnant women (Ooi et al. 2024; Robb et al. 2023) and diet quality indices developed for other countries may not be ideal for an Australian sample (Hodge and Bassett 2016). The DGI‐13 was therefore selected as it was developed for Australian adults and has been used to describe diet quality in Australian pregnant women previously (Dawson et al. 2021; Hill et al. 2021; van der Pligt et al. 2023).

The DGI‐13 includes 13 components scored from 0 to 10, with a maximum overall score of 130 (Thorpe et al. 2016). The components assessed dietary adequacy (intake of fruits, vegetables, grain and cereal foods [mostly wholegrain], dairy and dairy alternatives, lean meat and meat alternatives, and fluids), dietary variety, and moderation (limiting discretionary foods, saturated fat, unsaturated oils and spreads, added sugars and salt, and abstaining from alcohol). Usual daily servings of food groups (except for fruits and vegetables) were calculated by summing the daily equivalent servings for the relevant FFQ items (Thorpe et al. 2016). Fruit and vegetable servings were assessed via short dietary questions, as recommended for the DGI‐13 (Thorpe et al. 2016). Scoring for the alcohol component was altered for pregnancy, with any prenatal alcohol consumption receiving a score of zero.

### 2.4 Socioeconomic Factors

Socioeconomic factors assessed in this study included highest educational attainment, perceived social support, and stressful life events in the last 12 months (individual measures), as well as household income and perception of overall financial situation in the last 12 months (household level measures), and area‐level SES.

#### 2.4.1 Education

Participants self‐reported their highest educational attainment via a question sourced from the Australian Longitudinal Study on Women's Health (Australian Longitudinal Study on Women's Health n.d). Responses were combined into three groups for analysis: Less than Year 12; Year 12, Certificate, Diploma; and Bachelor degree or higher. Bachelor degree or above is commonly reported nationally in Australia (Australian Bureau of Statistics 2022a), while less than Year 12 (high school) may be indicative of disadvantage in pregnant women (Catherine et al. 2019).

#### 2.4.2 Perceived Social Support

The Multidimensional Scale of Perceived Social Support (MSPSS) (Zimet et al. 1988) was used to obtain a social support score (possible score range: 12–84). Higher scores indicate higher perceived support from a significant other, family, and friends.

#### 2.4.3 Stressful Life Events

Participants indicated if any of the following personal stressors had been a problem for them or someone close to them in the last 12 months: (1) Serious illness; (2) Serious accident; (3) Death of family member or close friend; (4) Mental illness; (5) Serious disability; (6) Loss of job or unable to get a job; (7) Discrimination; (8) Bullying and/or harassment; (9) Witness to violence; (10) Abuse or violent crime; (11) Trouble with the police; (12) Divorce or separation; (13) Alcohol or drug‐related problems; (14) Gambling problem; (15) Removal of children; (16) Being homeless; and (17) Being in prison/jail. The personal stressors list was obtained from the Australian Bureau of Statistics General Social Survey (Australian Bureau of Statistics 2021) and expanded for this study (items 16 and 17 were added; these experiences have been included in other studies of stressful life events in pregnancy (Mukherjee et al. 2017)). Latent class analysis (LCA) was used to identify unobserved groups (latent classes) based on response patterns to the 17 stressful life events. This classified pregnant women into subgroups with distinct types of stressors (further described in statistical analysis).

#### 2.4.4 Household Income

Participants selected the income range (AUD $0‐$25,999; $26,000–$51,999; $52,000–$103,999; $104,000–$207,999; ≥ $208,000) that best represented the total combined gross annual income of all household members they shared finances with. These income ranges have been used in previous Australian studies (e.g. Kerz et al. (2020)), and the wording of the question was informed by cognitive interviewing conducted by Bastian et al. (2022). To calculate equivalised income (income adjusted for household size and composition), a modified OECD factor (Australian Bureau of Statistics 2022b) was applied to the mid‐point of the income range. Equivalised income was then collapsed into quintiles. The first, third, and fifth quintiles were considered low, medium, and high income, respectively (Australian Bureau of Statistics 2022b).

#### 2.4.5 Overall Financial Situation

Perception of overall financial situation in the last 12 months was categorised as: ‘Spend more money than you get/cannot make ends meet’; ‘Just break even most months/just enough to make ends meet’; and ‘Able to save money most weeks/you are comfortable’. This question was sourced from the General Social Survey conducted by the Australian Bureau of Statistics (2021), and was modified to include additional wording based on a similar question used in a previous Australian study (Kerz et al. 2020). Overall financial situation was assessed in addition to income, as it considers other aspects such as expenses and debt, which may influence a household's food budget.

#### 2.4.6 Area‐Level SES

SEIFA Index of Relative Socioeconomic Advantage and Disadvantage (IRSAD) (Australian Bureau of Statistics 2023c) deciles were derived from self‐reported residential postcode and categorised as low (deciles 1–3), medium (4–7), and high (8–10) area‐level SES. Lower deciles indicate greater levels of socioeconomic disadvantage and lower levels of advantage in the area (vice versa for higher deciles).

### 2.5 Sociodemographic Variables

Participants self‐reported all other sociodemographic information, including age in years, number of previous births, marital status (data dichotomised to married/de facto vs*.* single/divorced/separated/widowed/in a relationship but not living together), and smoking status. Participants also self‐reported their pre‐pregnancy weight and height (used to calculate body mass index [BMI] in kg/m^2^). Pre‐pregnancy BMI was grouped into the categories used for monitoring and surveillance in Australia (< 18.5; 18.5–24.9; 25.0–29.9; ≥ 30.0) (Australian Bureau of Statistics 2023b).

### 2.6 Statistical Analysis

The analytic sample comprised participants with data available for the dependent variable (DGI‐13 score) and at least one of the socioeconomic measures. FFQ items with missing data were coded as ‘never or less than once per month’, if ≤ 10% of FFQ responses were missing. If > 10% of responses were missing, data were considered invalid, and the participant was excluded from the analytic sample (Thorpe et al. 2016). Missing data for socioeconomic measures were ≤ 5.6%. Analyses were conducted using the poLCA package (Linzer and Lewis 2011) in R version 4.4.1 for LCA, and IBM SPSS version 30 (IBM Corp 2024) for all other analyses. Statistical significance was considered *p* < 0.05.

#### 2.6.1 Latent Class Analysis

LCA was performed to classify pregnant women into subgroups with varying types of stressful life events. Missing data for stressful life events were minimal (5.4%) and unlikely to significantly impact modelling (Sinha et al. 2021); therefore, complete cases analysis was conducted. Models with increasing number of classes were run in a stepwise manner from one to six classes. To select the optimal model, the following were reviewed: Bayesian information criterion, which is commonly considered the most reliable model fit statistic (lower values indicate better fit) (Weller et al. 2020); Akaike information criterion (lower values indicate better fit, however this does not consider sample size and tends to reduce with increasing number of classes) (Sinha et al. 2021); and predicted class sizes. Interpretability was also considered in selecting the final model. Probabilities of belonging to each class were generated for each participant, and participants were then assigned to the class for which they had the highest probability. Probabilities of experiencing each stressor were also generated for each of the classes.

#### 2.6.2 Linear Regression

Multiple linear regression models examined associations between socioeconomic factors and overall diet quality score. For a multiple linear regression model with up to 12 independent variables, a minimum requirement of 360 participants was estimated (Wilson Van Voorhis and Morgan 2007). Over‐recruitment was performed to ensure sufficient representation across the spectrum of the socioeconomic measures and in consideration of the requirement for larger samples for LCA (Sinha et al. 2021). Confounding variables were determined a priori based on literature and theoretical considerations and minimum adjustment sets were identified via directed acyclic graphs (Figures S1 to S6 in Supporting Information) (Textor et al. 2016). Sensitivity analyses were conducted to confirm that the removal of outliers (defined as standardised residuals > 3) did not significantly alter model fit or estimates (i.e. < 10% change).

## 3 Results

### 3.1 Participants

Of the 2220 eligible respondents who consented and commenced the online survey, 640 (28.8%) did not complete the FFQ and were excluded from analyses (refer to Figure S7 in Supporting Information for a flow diagram summarising survey participation and exclusions). The analytic sample therefore comprised 1580 pregnant women, aged 17–41 years. Characteristics of the sample are presented in Table 1. Compared to survey respondents who did not complete the FFQ, participants in this sample were more likely to be university educated (Odds ratio [OR]: 1.4, 95% confidence interval [CI]: 1.2–1.7, *p* < 0.001) and to have a household income ≥ AUD $104,000 (OR: 1.5, 95% CI: 1.2–1.9, *p* < 0.001). They were also less likely to live in a low SES area (OR: 0.7, 95% CI: 0.5–0.9, *p* < 0.001) (refer to Table S3 in Supporting Information file S12).

**Table 1:** Characteristics of pregnant women (*n* = 1580).

	*n* (%)	Mean (SD)
Age (years)		30.6 (4.5)
Trimester of pregnancy		
First	315 (19.9)	
Second	721 (45.6)	
Third	544 (34.4)	
Pregnancy type		
Singleton	1556 (98.5)	
Twins	24 (1.5)	
Previous births		
0	880 (55.7)	
1	472 (29.9)	
> 1	228 (14.4)	
Children in household		
No	839 (53.1)	
Yes	741 (46.9)	
Born in Australia		
No	272 (17.4)	
Yes	1294 (82.6)	
Missing	14	
Aboriginal and/or Torres Strait Islander		
No	1534 (97.1)	
Yes	46 (2.9)	
Residential state		
Queensland	1534 (97.1)	
Other	46 (2.9)	
Education		
Less than Year 12	55 (3.5)	
Year 12, Certificate, Diploma	586 (37.1)	
Bachelor degree or higher	938 (59.4)	
Missing	1	
Married/de facto		
No	108 (6.9)	
Yes	1464 (93.1)	
Missing	8	
Household income (AUD)		
$0–$25,999	54 (3.5)	
$26,000–$51,999	118 (7.7)	
$52,000–$103,999	428 (27.8)	
$104,000–$207,999	717 (46.6)	
≥ $208,000	220 (14.3)	
Missing	43	
Equivalised household income		
Quintile 1 (low)	322 (20.9)	
Quintile 2	286 (18.6)	
Quintile 3 (middle)	387 (25.2)	
Quintile 4	369 (24.0)	
Quintile 5 (high)	173 (11.3)	
Missing	43	
Overall financial situation in the last 12 months		
Spend more money than you get/cannot make ends meet	90 (6.0)	
Just break even most weeks/just enough to make ends meet	478 (31.7)	
Able to save money most weeks/you are comfortable	940 (62.3)	
Missing	72	
Area‐level SES (IRSAD deciles)		
Low (1–3)	327 (20.8)	
Medium (4–7)	596 (37.9)	
High (8–10)	651 (41.4)	
Missing	6	
Pre‐pregnancy BMI (kg/m^2^)		
< 18.5	53 (3.5)	
18.5–24.9	739 (48.7)	
25.0–29.9	383 (25.2)	
≥ 30.0	344 (22.6)	
Missing	61	
Smoking status		
Non‐smoker	1543 (97.7)	
Smoker (any frequency)	37 (2.3)	
Received dietary advice in pregnancy so far		
No	685 (44.9)	
Yes	842 (55.1)	
Missing	53	
Taken supplements in pregnancy so far		
No	107 (6.8)	
Yes	1464 (93.2)	
Missing	9	
MSPSS score (possible score range:12‐84)		67.6 (15.6)^a^
SLEs in the last 12 months		
None	532 (35.6)	
1	363 (24.3)	
2	259 (17.3)	
≥ 3	341 (22.8)	
Missing	85	
DGI‐13 total score (max. score: 130)		76.1 (13.7)
DGI‐13 component score (max. score)		
Dietary variety (10)		4.6 (1.4)
Vegetables (10)		4.2 (2.3)
Fruit (10)		7.3 (3.2)
Grains and cereals (10)		4.0 (2.6)
Meat and alternatives (10)		7.2 (1.6)
Dairy and alternatives (10)		5.2 (2.9)
Fluids (10)		8.4 (2.0)
Limit discretionary foods (10)		3.2 (4.7)
Limit saturated fats (10)		5.6 (3.5)
Limit unsaturated fats (10)		9.6 (1.9)
Limit added salt (10)		4.3 (2.9)
Limit added sugars (10)		3.3 (4.7)
No alcohol (10)		9.4 (4.4)

^a^ *n* = 1490 due to missing data.

Abbreviations: AUD, Australian dollar; BMI, body mass index; DGI‐13, Dietary Guidelines Index 2013; IRSAD, Index of Relative Socioeconomic Advantage and Disadvantage; MSPSS, Multidimensional Scale of Perceived Social Support; SD, standard deviation; SES, socioeconomic status; SLEs, stressful life events.

### 3.2 Diet Quality

Mean prenatal diet quality was 76.1 (SD 13.7) out of a maximum possible score of 130. DGI‐13 component scores are reported in Table 1. Adherence to pregnancy dietary guidelines for core food groups (fruit; vegetables; grains and cereals; lean meats and alternatives; and dairy and dairy alternatives) was poor (Table 2). None of the participants consumed the minimum recommended daily servings for all five core food groups and over a third (35.0%) did not meet any of the core food group guidelines.

**Table 2:** Proportion of participants (*n* = 1580) adhering to dietary guidelines for pregnant women (National Health and Medical Research Council 2013).

Guideline (per day)	*n* (%) meeting guideline
Vegetables (≥ 5 servings)	56 (3.5)
Fruit (≥ 2 servings)	873 (55.3)
Grains and cereals (≥ 8.5 servings)	1 (0.1)
Lean meat and alternatives (≥ 3.5 servings)	217 (13.7)
Dairy and alternatives (≥ 2.5 servings)	241 (15.3)
Discretionary foods (≤ 2.5 servings)	503 (31.8)
Unsaturated spreads and oils (≤ 2 servings)	1518 (96.1)
Fluids (≥ 9 cups)	339 (21.5)
Alcohol (none in pregnancy)	1480 (93.7)

### 3.3 Socioeconomic Factors

Socioeconomic factors are reported in Table 1. Low equivalised income (quintile 1) equated to ≤ AUD $39,000, and high equivalised income (quintile 5) was ≥ AUD $115,560. Those with low equivalised income were more likely to indicate they either could not make ends meet or had just enough money to make ends meet most weeks (OR: 8.5, 95% CI: 6.4–11.5, *p* < 0.001). However, some participants in higher income households indicated this too; 31.0%, 16.9%, and 4.2% of those in quintiles 3 (middle income), 4, and 5 (high income), respectively.

**Figure 1:**
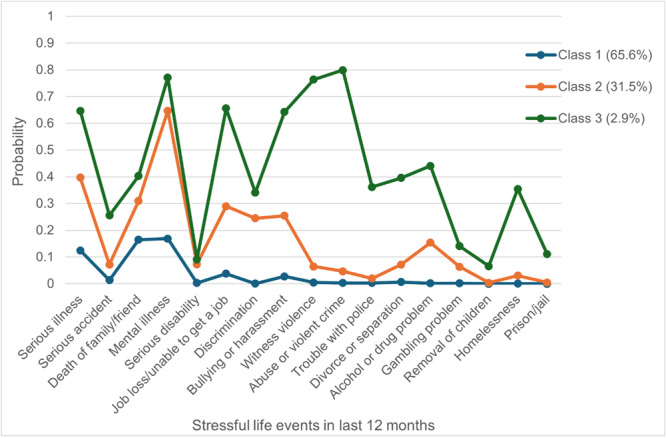
Latent classes of stressful life events in the last 12 months. Class 1, Minimal adversity; Class 2, Limited adversity; Class 3, Multi‐domain adversity.

In the LCA of stressful life events in the last 12 months, the Bayesian information criterion was lowest for the 3‐class model, however the Akaike information criterion continued to reduce as the number of classes increased (Table S4, file S13 and Figure S8 in Supporting Information). After consideration of model fit indices, class sizes (Table S4 in Supporting Information file S13) and interpretability, the 3‐class model was selected. The three latent classes are shown in Figure 1. More than half (*n* = 981/1495, 65.6%) of the participants were in class 1, which had a low probability of all the stressful life events and was named ‘minimal adversity’. Class 2 (*n* = 471/1495, 31.5%) had a high probability (64.7%) of mental illness, and a relatively high probability of employment issues, bullying, harassment, and discrimination, compared to class 1. Class 2 was considered ‘limited adversity’. Class 3 (*n* = 43/1495, 2.9%) had high probabilities (> 60%) of multiple stressors across various domains, such as physical and mental health, unemployment, bullying, harassment, and violence. Compared to the other classes, the probabilities of substance abuse, family, housing, and crime‐related issues were also relatively higher. It was therefore labelled ‘multi‐domain adversity’.

In the sample overall, the most common stressor endorsed by pregnant women in the last 12 months was mental illness (35.9%); in classes 1, 2, and 3 (minimal, limited, and multi‐domain adversity) the most prevalent stressor was death of a family member or close friend (18.1%), mental illness (71.5%), and abuse or violent crime (83.7%), respectively (Figure S9 in Supporting Information).

### 3.4 Socioeconomic Factors and Diet Quality

**Figure 2:**
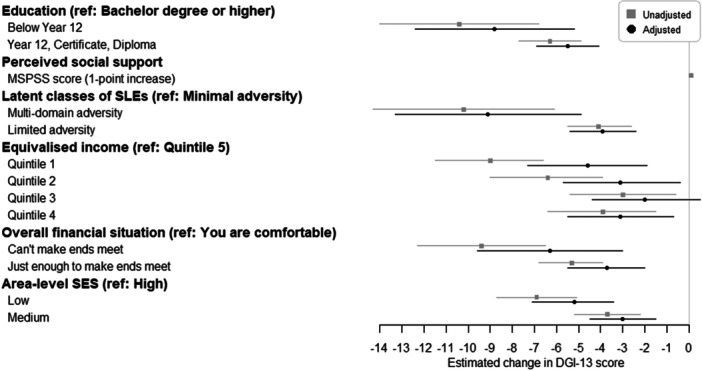
Forest plot of unadjusted and adjusted linear regression models examining associations between socioeconomic factors and diet quality score, measured by the Dietary Guidelines Index 2013 (DGI‐13). Boxes represent the beta‐coefficients from the unadjusted regression models; circles represent the beta‐coefficients from the adjusted regression models; lines show the 95% confidence intervals. The highest/most advantaged category was selected as the reference for each categorical socioeconomic factor. Confounding variables included in adjusted models: Education model adjusted for age; Latent classes of SLEs model adjusted for perceived social support; Equivalised income model adjusted for age, education level, and latent classes of SLEs; Overall financial situation model adjusted for equivalised income, perceived social support, and latent classes of SLEs; Area‐level SES model adjusted for equivalised income. DGI‐13, Dietary Guidelines Index 2013; MSPSS, Multidimensional Scale of Perceived Social Support; SES, socioeconomic status; SLEs, stressful life events.

All socioeconomic factors were significantly associated with diet quality score in unadjusted models, with estimates indicating a social gradient in diet quality (Figure 2 and Table S5 in Supporting Information file S14). After adjustment for confounders, the associations remained significant for all except the third quintile of equivalised income. There was some attenuation of estimates (see Figure 2 and Table S5 in Supporting Information file S14). One to three outliers were identified in each model, however sensitivity analyses indicated that these had minimal impact (< 6% change in beta‐coefficients).

## 4 Discussion

In this sample, prenatal diet quality was generally poor, and adherence to dietary guidelines was low. This is consistent with previous research in Australia (Maneschi et al. 2023; Slater et al. 2020; Wilkinson et al. 2022) and other high‐income countries (Rahmannia et al. 2024). Compared to Australian females aged 25–44 years, a higher proportion met the fruit recommendations (55.2% of the sample vs. 42.7% nationally) and a lower proportion met the vegetable recommendations (3.5% vs. 9.4%) (Australian Bureau of Statistics 2023a). Intakes of grain/cereal foods and vegetables were particularly low in our sample, with only 0.1% and 3.5% meeting the recommended daily servings for pregnant women (National Health and Medical Research Council 2013), respectively. These food groups are key contributors to folate/folic acid, iron, and iodine intakes in the Australian population (Australian Bureau of Statistics 2014). In pregnancy, these micronutrients are required in greater amounts and are important for foetal development (National Health and Medical Research Council 2006).

A social gradient in prenatal diet quality was evident with various socioeconomic factors measured at individual, household, and area levels. This is consistent with previous findings in a nationally representative sample of Australian adults, where lower education, household income, and area‐level SES were associated with lower diet quality (Livingstone et al. 2017). Of the socioeconomic factors assessed in the current study, education explained the most variation in prenatal diet quality and perceived social support explained the least, in the unadjusted models. In the adjusted models, the model for equivalised income (adjusted for age, education level, and latent classes of stressful life events) explained the most variation. The differences in diet quality were clinically relevant for all socioeconomic factors, except for perceived social support where a 10‐point score increase (indicating greater support) related to only 1‐point higher diet quality. For all other socioeconomic factors, the lowest or most disadvantaged categories (many of which are commonly used as proxies for low SES) were associated with reductions in diet quality of 5–9 points, and the middle categories (sometimes used as proxies for medium SES) were associated with a decrease of 3–6 points. A 9‐point lower DGI‐13 score could be the equivalent of missing 4.5 of the 5 recommended daily vegetable servings, for example, while a 3‐point decrease could be equivalent to consuming approximately 5 fewer daily servings of grain/cereal foods in pregnancy.

The associations observed between lower education and income with lower prenatal diet quality in this study are consistent with previous research conducted in other high‐income countries (Aubert et al. 2022; Doyle et al. 2017; Yu et al. 2022). In contrast, two recent Australian studies reported no association between education and prenatal diet quality score (Maneschi et al. 2023) or meeting food group recommendations (Wilkinson et al. 2022), or between income and meeting food group recommendations (Wilkinson et al. 2022). However, both were pilot studies with relatively small sample sizes and therefore may have been underpowered.

Compared to household income, perception of overall financial situation could theoretically be a better indicator of money available for adequate food, as it encompasses income, expenses, debt, and savings. While the estimates were reasonably similar between the models for overall financial situation and equivalised income, after adjustment for confounders the equivalised income model explained slightly more variation in diet quality. Nevertheless, this suggests a need to consider an individual's broader financial situation (i.e. even in the context of high income), particularly in the current cost‐of‐living crisis (Australian Bureau of Statistics 2025) where mortgage repayments and/or other expenses (including food) may have increased significantly without comparable increases in income.

To the authors' knowledge, the association observed in this study between different groups of stressors and prenatal diet quality is a novel contribution to the literature. Using LCA, a small number of participants were identified who were more likely to have recently (in the last 12 months) experienced stressors from multiple domains, including health, unemployment, bullying, harassment, violence, family, housing, and crime. Many of these stressors could increase the risk of food insecurity (Temple 2018). While this group was small (2.9% of the sample), the associated reduction in diet quality was substantial (9.1 points lower, compared to the minimal adversity group). The range of adverse experiences in this group suggests a greater likelihood of social disadvantage (Cohen et al. 2019); therefore, as people living with disadvantage are frequently underrepresented in research (Gallegos et al. 2023), a higher proportion of women could potentially be affected nationally.

While there is a need to improve diet quality among all pregnant women, this study provided evidence of a social gradient, suggesting that lower SES groups should be targeted as a priority. Failure to do so may exacerbate nutrition and health disparities, with significant implications for the health and wellbeing of future generations (Davies et al. 2016; McKerracher et al. 2020). Consideration of barriers and enablers specific to low SES groups, and inclusion of pregnant individuals with lived experience of disadvantage in future research, is necessary to ensure that any individual behaviour change interventions are not only efficacious among higher SES groups. For example, cognitive load is likely to be increased among those living with disadvantage and/or experiencing socioeconomic stressors, and should be considered in intervention design (Baxter et al. 2025). It is also essential that interventions beyond those grounded in individual responsibility and individual behaviour change are considered (such as upstream policy changes), given the structural barriers precluding many women from accessing a healthy diet. This could include social policies to ensure adequate income, particularly for casually employed or underemployed individuals without paid personal and/or parental leave entitlements, implementing screening for food and nutrition insecurity as part of routine antenatal care, and a food safety net programme for families with low income to increase access to healthy foods (no such programme currently exists in Australia).

In previous research on dietary inequities in Australia, income, education, and area‐level SES were used as proxies for SES most frequently (Lewis and Lee 2021). Careful consideration of the use of these (and other socioeconomic factors) as SES indicators in future research is recommended, as these variables are not interchangeable (Green and Popham 2019). The authors also stress that area‐level SES is not necessarily reflective of individual SES (Australian Bureau of Statistics 2023d). Area‐level SES may be associated with diet quality for reasons other than an individual's SES (e.g., differences in local food environments (Needham et al. 2022; Trapp et al. 2022)). It would therefore be prudent for future studies using area‐level measures of SES to highlight that these relate to attributes of the area or neighbourhood, rather than the individual.

The strengths of this study include the large sample size, the measurement of socioeconomic factors beyond income and education, and successful recruitment of participants from a range of socioeconomic backgrounds. The authors acknowledge that current SES does not necessarily reflect life course SES, which was not assessed in this study. It is also acknowledged that participation required time, literacy, and English language proficiency; therefore, the survey would have been inaccessible for some.

There are several limitations, including variability in the timeframes assessed by the different exposure variables (some referenced the last 12 months) and the outcome variable (the last 6 months). As usual dietary intake was assessed via a FFQ, recall and measurement error, and social desirability bias are likely (Lewis and Lee 2021; Naska et al. 2017). Caution should be exercised when drawing conclusions about proportions of participants consuming recommended daily servings of food groups, as assumed serving sizes were applied to convert the qualitative FFQ responses into DGI‐13 scores (with the exception of fruit and vegetables; refer to methods). While this is a significant limitation, these methods are in line with previous applications of the DGI‐13 where it was shown to demonstrate construct and convergent validity (McNaughton et al. 2008; Thorpe et al. 2016).

The generalisability of findings from this study may be somewhat limited, as convenience sampling was used to recruit participants (predominantly via social media advertisements that targeted women and mothers). Compared to pregnant women nationally, the participants in this study were similar in some respects such as average maternal age, proportion with a pre‐pregnancy BMI ≥ 30, and proportion who had a multiple pregnancy (Australian Institute of Health and Welfare 2024). However, more study participants were born in Australia (82.6% compared to 66.2% nationally) and a lower proportion identified as Aboriginal and/or Torres Strait Islander (2.9% vs. 5.2%) (Australian Institute of Health and Welfare 2024). Many other prenatal diet studies have not reported indigeneity, however, in most that have, even lower rates (≤ 1%) than the current study have been reported (Gallo et al. 2024; Pannu et al. 2024; Phillips et al. 2025). Over half (59.4%) of our study participants reported having a Bachelor degree or higher. Educational attainment is not regularly reported for pregnant women nationally; however, 50% of Australian females aged 25–44 years held a Bachelor degree or higher in 2022 (Australian Bureau of Statistics 2022a), suggesting that a higher proportion of our sample was university educated.

Compared to survey respondents who did not complete the FFQ, the study participants were more ‘advantaged’ (based on education, income, and area of residence). An underrepresentation of women living with disadvantage may have reduced the range and variability in diet quality scores in this sample, which could potentially contribute to weaker associations and attenuated estimates. Therefore, the social gradients in prenatal diet quality that were observed in this study may be more extreme in a more representative sample. While the findings of this study are not generalisable to all pregnant people, they contribute to understanding of the relationship between socioeconomic factors and prenatal diet quality in Australia and highlight a need to address dietary disparities.

## 5 Conclusion

While diet quality was poor overall in this sample of Australian pregnant women, various socioeconomic factors were significantly associated with DGI‐13 score. Lower equivalised income, education, and area‐level SES, not having enough money to be able to save regularly, as well as limited and multi‐domain adversity were all related to clinically important decreases in diet quality. These findings highlight the need for systems‐level interventions and policy change to reduce dietary and health inequities in pregnant women and their children.

## Author Contributions

B.W. contributed to the conceptualisation and design of the study, facilitated participant recruitment and data collection, undertook data analysis, and drafted the manuscript. D.G., S.N., and S.L.D. supervised the PhD candidate (B.W.) and contributed to the conceptualisation and design of the study, data interpretation, and critical revision of the manuscript. L.C., S.d.J., V.E., and A.K. contributed to participant recruitment and critical revision of the manuscript. All authors reviewed and approved the publication of the final version of the manuscript.

## Conflicts of Interest

The authors declare no conflicts of interest.

## Supporting information


**Figure S1:** Directed acyclic graph visualising the assumed relationship between education and prenatal diet quality. Exposure variable: education; Outcome variable: prenatal diet quality; Pink nodes: ancestor of exposure and outcome; Blue nodes: ancestor of outcome.

**Figure S2:** Directed acyclic graph visualising the assumed relationship between perceived social support and prenatal diet quality. Exposure variable: perceived social support; Outcome variable: prenatal diet quality; Green nodes: ancestor of exposure; Blue nodes: ancestor of outcome.

**Figure S3:** Directed acyclic graph visualising the assumed relationship between latent classes of stressful life events (SLEs) in the last 12 months and prenatal diet quality. Exposure variable: latent classes of SLEs in the last 12 months; Outcome variable: prenatal diet quality; Pink nodes: ancestor of exposure and outcome; Blue nodes: ancestor of outcome.

**Figure S4:** Directed acyclic graph visualising the assumed relationship between equivalised income and prenatal diet quality. Exposure variable: equivalised income; Outcome variable: prenatal diet quality; Pink nodes: ancestor of exposure and outcome; Green nodes: ancestor of exposure; Blue nodes: ancestor of outcome.

**Figure S5:** Directed acyclic graph visualising the assumed relationship between perception of overall financial situation in the last 12 months and prenatal diet quality. Exposure variable: overall financial situation in the last 12 months; Outcome variable: prenatal diet quality; Pink nodes: ancestor of exposure and outcome; Blue nodes: ancestor of outcome.

**Figure S6:** Directed acyclic graph visualising the assumed relationship between area‐level SES and prenatal diet quality. Exposure variable: area SES; Outcome variable: prenatal diet quality; Pink nodes: ancestor of exposure and outcome; Blue nodes: ancestor of outcome.

**Figure S7:** Flow diagram summarising online survey participation between August 2022 and March 2023 by Australian pregnant women and eligibility for inclusion in the analytic sample (*n* = 1,580).

**Figure S8:** Elbow plot of fit indices for latent class analysis of stressful life events in the last 12 months.

**Figure S9.** Prevalence of stressors by latent classes of stressful life events in the last 12 months. Class 1: Minimal adversity; Class 2: Limited adversity; Class 3: Multi‐domain adversity. The percentages in the legend represent the proportion of the sample in the class.

**Table S1:** STROBE Statement—Checklist of items that should be included in reports of cross‐sectional studies (von Elm et al. ; 2007).

**Table S2:** The Checklist for Reporting Results of Internet E‐Surveys (CHERRIES) (Eysenbach, 2004).

**Table S3:** Sociodemographic characteristics of the analytic sample of 1,580 pregnant women, compared to survey respondents who did not complete the food frequency questionnaire.

**Table S4:** Model fit indices and results for latent class analysis of stressful life events in the last 12 months.

**Table S5.** Unadjusted and adjusted associations of socioeconomic factors with diet quality, measured by the Dietary Guidelines Index 2013 (DGI‐13).

## Data Availability

The data that support the findings of this study are available from the corresponding author upon reasonable request.
