# Supplementary material for: Socioeconomic Factors Associated With Diet Quality in Pregnancy: A Cross‐Sectional Australian Study
Source: Matern Child Nutr. 2026 Feb 12;22(1):e70170. doi: 10.1111/mcn.70170 (PMC12896378; doi:10.1111/mcn.70170)
Supplement: Supplementary file 2 — Figure S2: Directed acyclic graph visualising the assumed relationship between perceived social support and prenatal diet quality. Exposure variable: perceived social support; Outcome variable: prenatal diet quality; Green nodes: ancestor of exposure; Blue nodes: ancestor of outcome. [file MCN-22-e70170-s009.docx]

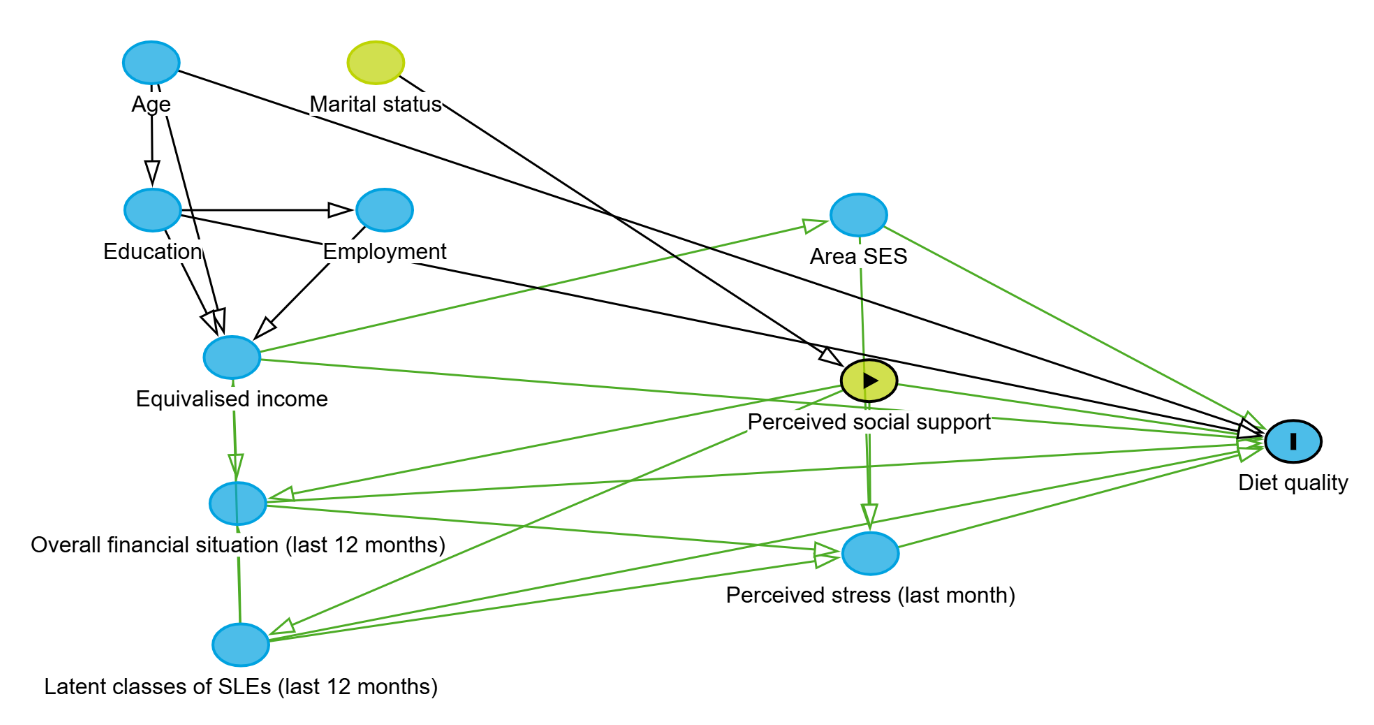


**Figure S2**. Directed acyclic graph visualising the assumed relationship between perceived social support and prenatal diet quality. Exposure variable: perceived social support; Outcome variable: prenatal diet quality; Green nodes: ancestor of exposure; Blue nodes: ancestor of outcome.

Abbreviations: SES, Socioeconomic status; SLEs, Stressful life events.
