# Supplementary material for: Socioeconomic Factors Associated With Diet Quality in Pregnancy: A Cross‐Sectional Australian Study
Source: Matern Child Nutr. 2026 Feb 12;22(1):e70170. doi: 10.1111/mcn.70170 (PMC12896378; doi:10.1111/mcn.70170)
Supplement: Supplementary file 4 — Figure S4: Directed acyclic graph visualising the assumed relationship between equivalised income and prenatal diet quality. Exposure variable: equivalised income; Outcome variable: prenatal diet quality; Pink nodes: ancestor of exposure and outcome; Green nodes: ancestor of exposure; Blue nodes: ancestor of outcome. [file MCN-22-e70170-s008.docx]

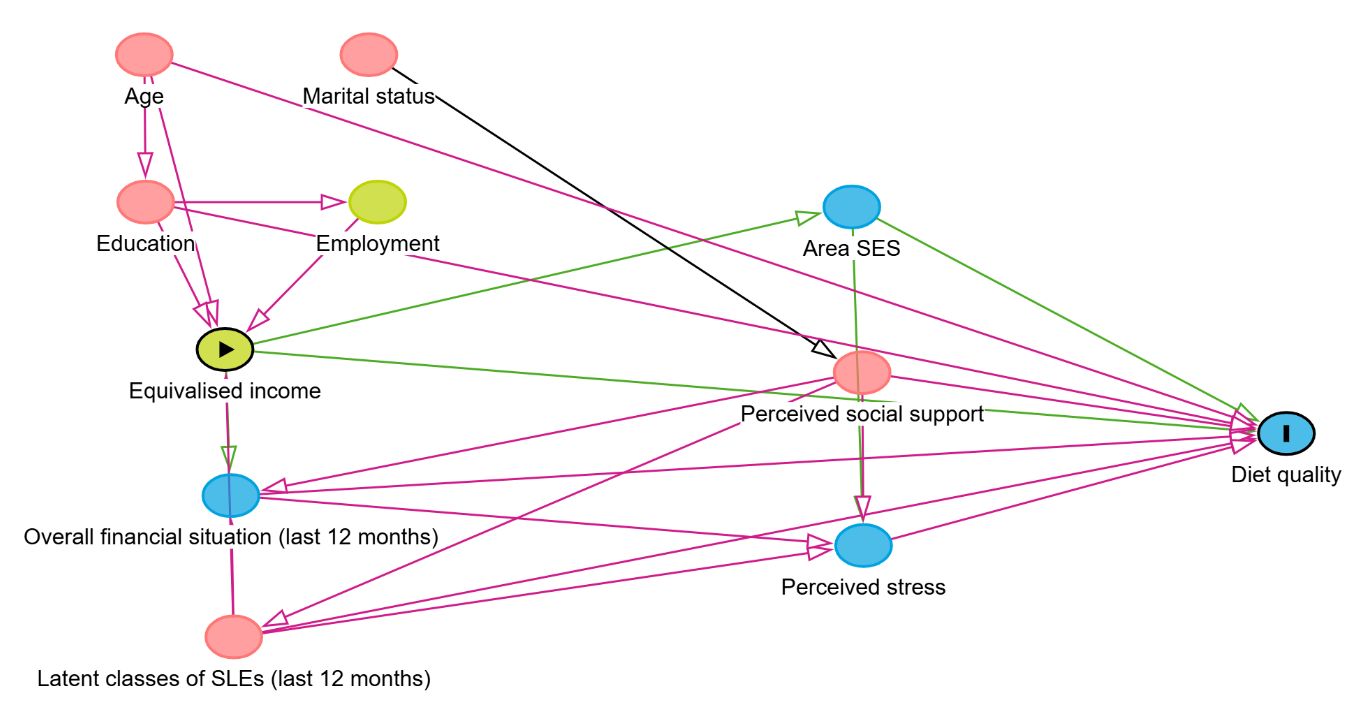


**Figure S4**. Directed acyclic graph visualising the assumed relationship between equivalised income and prenatal diet quality. Exposure variable: equivalised income; Outcome variable: prenatal diet quality; Pink nodes: ancestor of exposure and outcome; Green nodes: ancestor of exposure; Blue nodes: ancestor of outcome.

Abbreviations: SES, Socioeconomic status; SLEs, Stressful life events.
