# Supplementary material for: Socioeconomic Factors Associated With Diet Quality in Pregnancy: A Cross‐Sectional Australian Study
Source: Matern Child Nutr. 2026 Feb 12;22(1):e70170. doi: 10.1111/mcn.70170 (PMC12896378; doi:10.1111/mcn.70170)
Supplement: Supplementary file 7 — Figure S7: Flow diagram summarising online survey participation between August 2022 and March 2023 by Australian pregnant women and eligibility for inclusion in the analytic sample (n = 1,580). [file MCN-22-e70170-s013.docx]

***n* = 122** consented to participate but did not commence the survey

***n* = 574** did not consent to participate

***n* = 54** were ineligible to participate based on screening questions

***n* = 2,220** participants included after data integrity checks

**Participant recruitment**

***n* = 2,282** participants commenced the survey

***n* = 2,404** included after the consent process

***n* = 2,978** self-identified as eligible to participate

***n* = 3,032** accessed the online survey screening questions via an advertisement

***n* = 62** responses with unreliable or ineligible data

- *n* = 61 duplicate responses
- *n* = 1 reCAPTCHA score < 0.5

**Data cleaning**

***n* = 640** did not complete the FFQ to determine diet quality score

**Data analysis**

***n* = 1,580** participants included in analyses

**Figure S7**. Flow diagram summarising online survey participation between August 2022 and March 2023 by Australian pregnant women and eligibility for inclusion in the analytic sample (*n* = 1,580)

Abbreviations: FFQ, Food frequency questionnaire.
