# Supplementary material for: Socioeconomic Factors Associated With Diet Quality in Pregnancy: A Cross‐Sectional Australian Study
Source: Matern Child Nutr. 2026 Feb 12;22(1):e70170. doi: 10.1111/mcn.70170 (PMC12896378; doi:10.1111/mcn.70170)
Supplement: Supplementary file 8 — Figure S8: Elbow plot of fit indices for latent class analysis of stressful life events in the last 12 months. [file MCN-22-e70170-s006.docx]

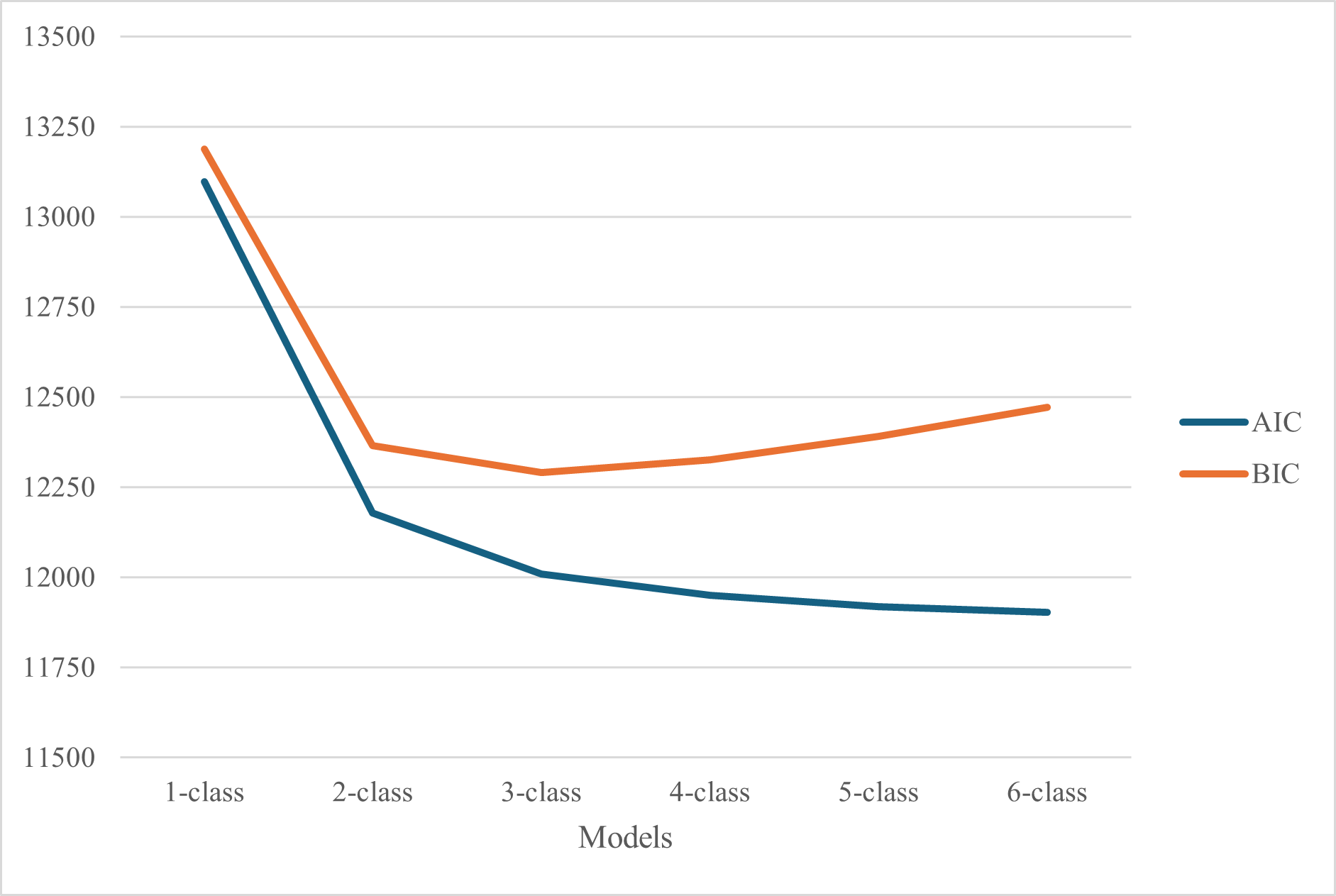


**Figure S8**. Elbow plot of fit indices for latent class analysis of stressful life events in the last 12 months.

Abbreviations: AIC, Akaike information criterion; BIC, Bayesian information criterion.
