# Supplementary material for: Socioeconomic Factors Associated With Diet Quality in Pregnancy: A Cross‐Sectional Australian Study
Source: Matern Child Nutr. 2026 Feb 12;22(1):e70170. doi: 10.1111/mcn.70170 (PMC12896378; doi:10.1111/mcn.70170)
Supplement: Supplementary file 9 — Figure S9. Prevalence of stressors by latent classes of stressful life events in the last 12 months. Class 1: Minimal adversity; Class 2: Limited adversity; Class 3: Multi‐domain adversity. The percentages in the legend represent the proportion of the sample in the class. [file MCN-22-e70170-s005.docx]

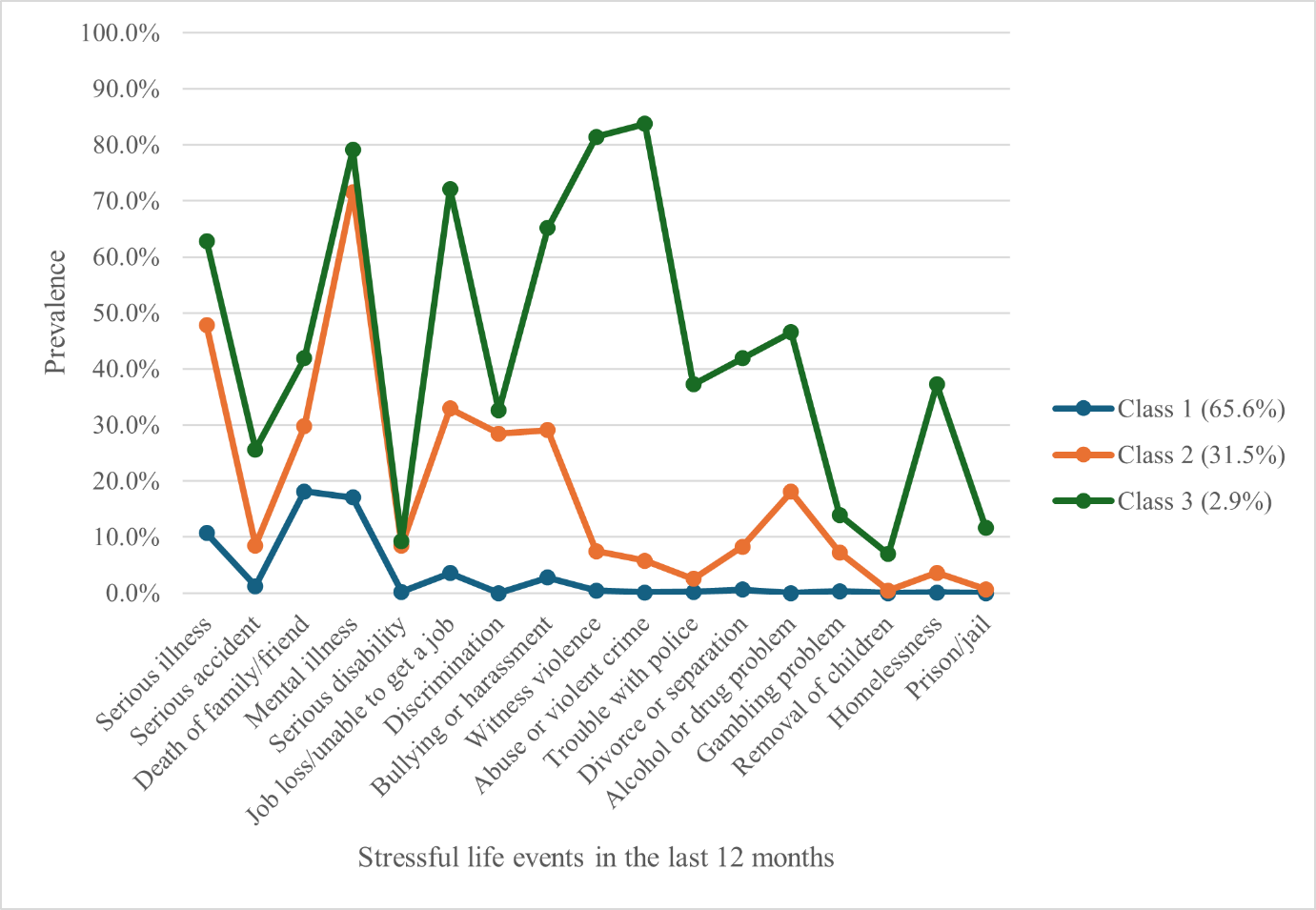


**Figure S9.** Prevalence of stressors by latent classes of stressful life events in the last 12 months. Class 1: Minimal adversity; Class 2: Limited adversity; Class 3: Multi-domain adversity. The percentages in the legend represent the proportion of the sample in the class.
