# Supplementary material for: Socioeconomic Factors Associated With Diet Quality in Pregnancy: A Cross‐Sectional Australian Study
Source: Matern Child Nutr. 2026 Feb 12;22(1):e70170. doi: 10.1111/mcn.70170 (PMC12896378; doi:10.1111/mcn.70170)
Supplement: Supplementary file 11 — Table S2: The Checklist for Reporting Results of Internet E‐Surveys (CHERRIES) (Eysenbach, 2004). [file MCN-22-e70170-s007.docx]

**Table S2:** The Checklist for Reporting Results of Internet E-Surveys (CHERRIES) (Eysenbach, 2004)

| Item Category | Checklist Item(s) | Checklist Item(s) Explanation | Page Number | Additional Information |
| --- | --- | --- | --- | --- |
| Design | Describe survey design | Describe target population, sample frame. Is the sample a convenience sample? (In “open” surveys this is most likely.) | 5 | The sample was a convenience sample. |
| IRB (Institutional Review Board) approval and informed consent process | IRB approval  Informed consent  Data protection | Mention whether the study has been approved by an IRB.  Describe the informed consent process. Were the participants told the length of time of the survey, which data were stored and where and for how long, who the investigator was, and the purpose of the study?  If any personal information was collected or stored, describe what mechanisms were used to protect unauthorised access. | 6 | Participants were provided with a participant information statement and the estimated time required to complete the survey before the consent process. The participant information statement listed the study investigators and provided information on the purpose of the survey and the use, storage and protection of personal information and data.  After accessing the participant information statement, participants were asked if they consented to take part and were required to click ‘Yes, I consent’ to confirm this before they could proceed to the online survey. |
| Development and pre-testing | Development and testing | State how the survey was developed, including whether the usability and technical functionality of the electronic questionnaire had been tested before fielding the questionnaire. | 5 | The survey was developed in Qualtrics using pre-existing tools to assess usual dietary intake , perceived social support, and stressful life events. Other sociodemographic questions were sourced from national surveys conducted in Australia.  Pilot testing of the survey was conducted among five colleagues and/or people similar to the target group (i.e. women of reproductive age or pregnant women). Feedback was sought on the time required for completion, whether there were any questions that were difficult to understand or answer, or if any of the questions raised concerns.  Testing of the functionality of the survey (i.e. adaptive questioning) was also carried out by research team members and other colleagues. |
| Recruitment process and description of the sample having access to the questionnaire | Open survey versus closed survey  Contact mode  Advertising the survey | An “open survey” is a survey open for each visitor of a site, while a closed survey is only open to a sample which the investigator knows (password-protected survey).  Indicate whether or not the initial contact with the potential participants was made on the Internet. (Investigators may also send out questionnaires by mail and allow for Web-based data entry.)  How/where was the survey announced or advertised? Some examples are offline media (newspapers), or online (mailing lists – If yes, which ones?) or banner ads (Where were these banner ads posted and what did they look like?). It is important to know the wording of the announcement as it will heavily influence who chooses to participate. Ideally, the survey announcement should be published as an appendix. | 5-6 | The survey was open, with no password required to enter.  Advertisements were displayed electronically via social media (Facebook and Instagram) and physically at antenatal clinics. Advertisements stated: ‘we’re running an important study to understand how difficulty making ends meet relates to diet quality in pregnancy’ and/or asked participants to ‘help us learn how we can support fair access to food for all women/families during pregnancy by participating in this research study’. Advertisements also included ‘to thank you for participating in the survey, we are offering entry into a draw to win 1 of 3 $200 gift cards’ or similar. |
| Survey administration | Web/e-mail  Context  Mandatory/voluntary  Incentives  Time/date  Randomization of items or questionnaires  Adaptive questioning  Number of items  Number of screens (pages)  Completeness check  Review step | State the type of e-survey (e.g., one posted on a Web site, or one sent out through e-mail). If it is an e-mail survey, were the responses entered manually into a database, or was there an automatic method for capturing responses?  Describe the website (for mailing list/newsgroup) in which the survey was posted. What is the website about, who is visiting it, what are visitors normally looking for? Discuss to what degree the content of the website could pre-select the sample or influence the results. For example, a survey about vaccination on an anti-immunisation website will have different results from a web survey conducted on a government website  Was it a mandatory survey to be filled in by every visitor who wanted to enter the website, or was it a voluntary survey?  Were any incentives offered (e.g., monetary, prizes, or non-monetary incentives such as an offer to provide the survey results)?  In what timeframe were the data collected?  To prevent biases, items can be randomised or alternated.  Use adaptive questioning (certain items, or only conditionally displayed based on responses to other items) to reduce the number and complexity of the questions.  What was the number of questionnaire items per page? The number of items is an important factor for the completion rate.  Over how many pages was the questionnaire distributed? The number of items is an important factor for the completion rate.  It is technically possible to do consistency or completeness checks before the questionnaire is submitted. Was this done, and if “yes”, how (usually JAVAScript)? An alternative is to check for completeness after the questionnaire has been submitted (and highlight mandatory items). If this has been done, it should be reported. All items should provide a non-response option, such as “not applicable” or “rather not say”, and selection of one response option should be enforced.  State whether respondents were able to review and change their answers (e.g., through a Back button or a Review step which displays a summary of the responses and asks the respondents if they are correct). | 5-6 | The survey was voluntary and was hosted on Qualtrics. To enter the survey, participants had to click on the electronic advertisement or scan a QR code. Participants were offered entry in a prize draw (randomly selected) to win one of three AUD $200 gift cards. Participants could also indicate if they wished to receive a copy of the overall results of the study after it has finished.  Items were not randomised. Adaptive questioning was used.  The number of items per page varied. A maximum of 244 questions over 35 web pages were asked (for some participants, this was reduced by adaptive questioning).  Most questions in the survey were mandatory to respond to (with a response option of either ‘don’t know’ or ‘prefer not to say’) and the respondents were unable to move forward in the survey without completing them. However, some questions were not mandatory to respond to. Where responses were not mandatory, participants received a reminder/prompt to answer them before continuing to the next question.  Participants could click the “Back” button, enabling them to change their responses. Incomplete surveys could be continued by the respondent for up to one week from their last response/action. After this time, the data in the incomplete survey was automatically recorded by Qualtrics and the participant could no longer edit or continue that response. |
| Response rates | Unique site visitor  view rate (ratio of unique survey visitors/unique site visitors)  Participation rate (Ratio of unique visitors who agreed to participate/unique first survey page visitors)  Completion rate (Ratio of users who finished the survey/users who agreed to participate) | If you provide view rates or participation rates, you need to define how you determined a unique visitor. There are different techniques available, based on IP addresses, cookies or both.  Requires counting unique visitors to the first page of the survey, divided by the number of unique site visitors (not page views!). It is not unusual to have view rates of less than 0.1 % if the survey is voluntary.  Count the unique number of people who filled in the first survey page (or agreed to participate, for example by checking a checkbox), divided by visitors who visit the first page of the survey (or the informed consents page, if present). This can also be called “recruitment” rate.  The number of people submitting the last questionnaire page, divided by the number of people who agreed to participate (or submitted the first survey page). This is only relevant if there is a separate “informed consent” page or if the survey goes over several pages. This is a measure for attrition. Note that “completion” can involve leaving questionnaire items blank. This is not a measure for how completely questionnaires were filled in. (If you need a measure for this, use the word “completeness rate”.) | Supplementary Figure S7 | Response rate could not be calculated due to the method of survey distribution. Paid Meta advertising (Facebook and Instagram) generated 3,210 clicks and 177,856 impressions in total.  Of the eligible survey respondents who consented and commenced the survey (*n* = 2,220), 92.5% were recruited via paid Meta advertising, 5% were recruited via unpaid Facebook advertisements (posts), and 2.2% were recruited via flyers/posters at local public antenatal clinics.  Of the analytic sample (*n* = 1,580), 92.0% were recruited via paid Meta advertising, 5.3% were recruited via unpaid Facebook advertisements, and 2.4% were recruited via flyers/posters at local antenatal clinics. |
| Preventing multiple entries from the same individual | Cookies used  IP check  Log file analysis  Registration | Indicate whether cookies were used to assign a unique user identifier to each client computer. If so, mention the page on which the cookie was set and read, and how long the cookie was valid. Were duplicate entries avoided by preventing users access to the survey twice; or were duplicate database entries having the same user ID eliminated before analysis? In the latter case, which entries were kept for analysis (e.g., the first entry or the most recent)?  Indicate whether the IP address of the client computer was used to identify potential duplicate entries from the same user. If so, mention the period of time for which no two entries from the same IP address were allowed (e.g., 24 hours). Were duplicate entries avoided by preventing users with the same IP address access to the survey twice; or were duplicate database entries having the same IP address within a given period of time eliminated before analysis? If the latter, which entries were kept for analysis (e.g., the first entry or the most recent)?  Indicate whether other techniques to analyze the log file for identification of multiple entries were used. If so, please describe.  In “closed” (non-open) surveys, users need to login first and it is easier to prevent duplicate entries from the same user. Describe how this was done. For example, was the survey never displayed a second time once the user had filled it in, or was the username stored together with the survey results and later eliminated?  If the latter, which entries were kept for analysis (eg, the first entry or the most recent)? | Supplementary Figure S7 | To flag potential duplicate responses, IP address was used. Responses recorded with the same IP address were then reviewed manually to determine if they were duplicates. This included a review of contact information (provided for prize draw entry and/or expression of interest for participating in a related research study), sociodemographic variables, and identification of any other suspicious information, as it is possible for the same IP address to be recorded for different people. Where duplicates were identified, the most complete response was kept. If both responses were complete, the most recent response was kept.  Other checks for duplicates included review of contact information data. |
| Analysis | Handling of incomplete questionnaires  Questionnaires submitted with an atypical timestamp  Statistical correction | Were only completed questionnaires analyzed? Were questionnaires which terminated early (where, for example, users did not go through all questionnaire pages) also analyzed?  Some investigators may measure the time people needed to fill in a questionnaire and exclude questionnaires that were submitted too soon. Specify the timeframe that was used as a cut-off point, and describe how this point was determined.  Indicate whether any methods such as weighting of items or propensity scores have been used to adjust for the non-representative sample; if so, please describe the methods. | 11; Supplementary Figure S7 | Incomplete surveys were analysed if the participant provided data for the outcome variable (diet quality score) and at least one of the socioeconomic factors measured in this study.  Survey completion times were reviewed to identify if any participants had completed the survey > 2 standard deviations below the median duration and/or less than one-third of the median duration. None were identified.  Methods such as weighting of items or propensity scores were not used. |

**References**

Eysenbach, G. (2004). Improving the quality of web surveys: The Checklist for Reporting Results of Internet E-Surveys (CHERRIES). *Journal of Medical Internet Research*, *6*(3), e34. <https://doi.org/10.2196/jmir.6.3.e34>
