# Supplementary material for: Socioeconomic Factors Associated With Diet Quality in Pregnancy: A Cross‐Sectional Australian Study
Source: Matern Child Nutr. 2026 Feb 12;22(1):e70170. doi: 10.1111/mcn.70170 (PMC12896378; doi:10.1111/mcn.70170)
Supplement: Supplementary file 12 — Table S3: Sociodemographic characteristics of the analytic sample of 1,580 pregnant women, compared to survey respondents who did not complete the food frequency questionnaire. [file MCN-22-e70170-s001.docx]

**Table S3.** Sociodemographic characteristics of the analytic sample of 1,580 pregnant women**,** compared to survey respondents who did not complete the food frequency questionnaire

|  | Mean (SD) or *n* (%) | |  |
| --- | --- | --- | --- |
|  | Analytic sample  (*n* = 1,580) | Respondents not included in analyses^a^ | *P* value^b^ |
| Age (years) | 30.6 (4.5) | 29.7 (4.9)^c^ | < 0.001 |
| Trimester of pregnancy |  |  | 0.281 |
| First | 315 (19.9) | 146 (22.8) |  |
| Second | 721 (45.6) | 275 (43.0) |  |
| Third | 544 (34.4) | 219 (34.2) |  |
| Pregnancy type |  |  | 0.273 |
| Singleton | 1,556 (98.5) | 632 (98.8) |  |
| Twins | 24 (1.5) | 7 (1.1) |  |
| Triplets or more | 0 (0) | 1 (0.2) |  |
| Previous births |  |  | 0.455 |
| 0 | 880 (55.7) | 340 (53.1) |  |
| 1 | 472 (29.9) | 208 (32.5) |  |
| > 1 | 228 (14.4) | 92 (14.4) |  |
| Children in household |  |  | 0.061 |
| No | 839 (53.1) | 247 (48.3) |  |
| Yes | 741 (46.9) | 264 (51.7) |  |
| Missing | 0 | 129 |  |
| Born in Australia |  |  | 0.783 |
| No | 272 (17.4) | 109 (17.9) |  |
| Yes | 1,294 (82.6) | 501 (82.1) |  |
| Missing | 14 | 30 |  |
| Aboriginal and/or Torres Strait Islander |  |  | 0.055 |
| No | 1,534 (97.1) | 586 (95.4) |  |
| Yes | 46 (2.9) | 28 (4.6) |  |
| Missing | 0 | 26 |  |
| Married / De facto |  |  | 0.095 |
| No | 108 (6.9) | 55 (9.0) |  |
| Yes | 1,464 (93.1) | 559 (91.0) |  |
| Missing | 8 | 26 |  |
| Education |  |  | 0.001 |
| Less than Year 12 | 55 (3.5) | 25 (4.1) |  |
| Year 12, Certificate, Diploma | 586 (37.1) | 278 (45.1) |  |
| Bachelor degree or higher | 938 (59.4) | 313 (50.8) |  |
| Missing | 1 | 24 |  |
| Household income (AUD) |  |  | < 0.001 |
| $0-$25,999 | 54 (3.5) | 27 (6.5) |  |
| $26,000-$51,999 | 118 (7.7) | 48 (11.5) |  |
| $52,000-$103,999 | 428 (27.8) | 129 (30.9) |  |
| $104,000-$207,999 | 717 (46.6) | 168 (40.2) |  |
| ≥$208,000 | 220 (14.3) | 46 (11.0) |  |
| Missing | 43 | 222 |  |
| Overall financial situation in the last 12 months |  |  | 0.063 |
| Spend more money than you get / cannot make ends meet | 90 (6.0) | 26 (6.3) |  |
| Just break even most weeks / just enough to make ends meet | 478 (31.7) | 156 (37.6) |  |
| Able to save money most weeks / you are comfortable | 940 (62.3) | 233 (56.1) |  |
| Missing | 72 | 225 |  |
| Area-level SES (IRSAD deciles^d^) |  |  | 0.001 |
| Low (1-3) | 327 (20.8) | 145 (27.8) |  |
| Medium (4-7) | 596 (37.9) | 198 (38.0) |  |
| High (8-10) | 651 (41.4) | 178 (34.2) |  |
| Missing | 6 | 119 |  |
| Pre-pregnancy BMI (kg/m^2^) |  |  | 0.488 |
| < 18.5 | 53 (3.5) | 19 (3.6) |  |
| 18.5-24.9 | 739 (48.7) | 248 (47.6) |  |
| 25.0-29.9 | 383 (25.2) | 120 (23.0) |  |
| ≥ 30.0 | 344 (22.6) | 134 (25.7) |  |
| Missing | 61 | 119 |  |
| Smoking status |  |  | 0.290 |
| Non-smoker | 1,543 (97.7) | 586 (96.9) |  |
| Smoker (any frequency) | 37 (2.3) | 19 (3.1) |  |
| Missing | 0 | 35 |  |

^a^ Maximum of *n* = 640. *N* varies for each variable, depending on when respondents dropped out of the survey.

^b^ *P* values derived from an independent samples *t*-test for age (continuous variable) and ꭓ^2^ tests or Fisher’s exact tests (if assumptions for ꭓ^2^ test were violated) for all other variables (categorical).

^c^ *n* = 622

^d^ IRSAD deciles were derived from self-reported residential postcode, as an indicator of area-level socioeconomic status. Lower deciles reflect higher relative socioeconomic disadvantage and lower relative advantage in the area (vice versa for higher deciles).

Abbreviations: AUD, Australian dollars; BMI, Body mass index; IRSAD, Index of Relative Socioeconomic Advantage and Disadvantage; SES, Socioeconomic status; SD, Standard deviation.
