# Supplementary material for: Socioeconomic Factors Associated With Diet Quality in Pregnancy: A Cross‐Sectional Australian Study
Source: Matern Child Nutr. 2026 Feb 12;22(1):e70170. doi: 10.1111/mcn.70170 (PMC12896378; doi:10.1111/mcn.70170)
Supplement: Supplementary file 13 — Table S4: Model fit indices and results for latent class analysis of stressful life events in the last 12 months. [file MCN-22-e70170-s010.docx]

**Table S4.** Model fit indices and results for latent class analysis of stressful life events in the last 12 months

| Model | Log-likelihood | AIC | BIC | Smallest predicted class size |
| --- | --- | --- | --- | --- |
| 1-class | -6,531.457 | 13,096.91 | 13,187.18 |  |
| 2-class | -6,054.628 | 12,179.26 | 12,365.1 | 22.2% |
| 3-class | -5,951.111 | 12,008.22 | **12,289.65** | 2.9% |
| 4-class | -5,903.771 | 11,949.54 | 12,326.54 | 2.4% |
| 5-class | -5,870.472 | 11,918.94 | 12,391.52 | 1.2% |
| 6-class | -5,844.506 | 11,903.01 | 12,471.17 | 1.3% |

Abbreviations: AIC, Akaike information criterion; BIC, Bayesian information criterion.
