# Supplementary material for: Socioeconomic Factors Associated With Diet Quality in Pregnancy: A Cross‐Sectional Australian Study
Source: Matern Child Nutr. 2026 Feb 12;22(1):e70170. doi: 10.1111/mcn.70170 (PMC12896378; doi:10.1111/mcn.70170)
Supplement: Supplementary file 14 — Table S5. Unadjusted and adjusted associations of socioeconomic factors with diet quality, measured by the Dietary Guidelines Index 2013 (DGI‐13). [file MCN-22-e70170-s004.docx]

**Table S5.** Unadjusted and adjusted associations of socioeconomic factors with diet quality, measured by the Dietary Guidelines Index 2013 (DGI-13)

|  | n | Unadjusted β  (95% CI) | *P* value | n | Adjusted β  (95% CI) | *P* value |
| --- | --- | --- | --- | --- | --- | --- |
| Education | 1,579 | Adj R^2^ = 0.059 |  | 1,579 | Adj R^2^ = 0.075 |  |
| Below Year 12 | 55 | -10.4 (-14.0, -6.8) | < 0.001 | 55 | -8.8 (-12.4, -5.2)^a^ | < 0.001 |
| Year 12, Certificate, or Diploma | 586 | -6.3 (-7.7, -4.9) | < 0.001 | 586 | -5.5 (-6.9, -4.1)^a^ | < 0.001 |
| Bachelor degree or higher | 938 | Reference |  | 938 | Reference |  |
| Perceived social support | 1,490 | Adj R^2^ = 0.010 |  |  |  |  |
| 1-point increase in score | 1,490 | 0.1 (0.04, 0.13) | < 0.001 |  |  |  |
| Latent classes of SLEs | 1,495 | Adj R^2^ = 0.030 |  | 1,485 | Adj R^2^ = 0.034 |  |
| Multi-domain adversity | 43 | -10.2 (-14.3, -6.1) | < 0.001 | 43 | -9.1 (-13.3, -4.9)^b^ | < 0.001 |
| Limited adversity | 471 | -4.1 (-5.5, -2.6) | < 0.001 | 470 | -3.9 (-5.4, -2.4)^b^ | < 0.001 |
| Minimal adversity | 981 | Reference |  | 972 | Reference |  |
| Equivalised income | 1,537 | Adj R^2^ = 0.040 |  | 1,455 | Adj R^2^ = 0.091 |  |
| Quintile 1 (low) | 322 | -9.0 (-11.5, -6.6) | < 0.001 | 297 | -4.6 (-7.3, -1.9)^c^ | < 0.001 |
| Quintile 2 | 286 | -6.4 (-9.0, -3.9) | < 0.001 | 271 | -3.1 (-5.7, -0.4)^c^ | 0.022 |
| Quintile 3 (medium) | 387 | -3.0 (-5.4, -0.6) | 0.015 | 369 | -2.0 (-4.4, 0.5)^c^ | 0.111 |
| Quintile 4 | 369 | -3.9 (-6.4, -1.5) | 0.001 | 356 | -3.1 (-5.5, -0.7)^c^ | 0.012 |
| Quintile 5 (high) | 173 | Reference |  | 162 | Reference |  |
| Perception of overall financial situation | 1,508 | Adj R^2^ = 0.048 |  | 1,388 | Adj R^2^ = 0.073 |  |
| Spend more money than you get / can’t make ends meet | 90 | -9.4 (-12.3, -6.5) | < 0.001 | 81 | -6.3 (-9.6, -3.0)^d^ | < 0.001 |
| Just break even most weeks / just enough to make ends meet | 478 | -5.3 (-6.8, -3.9) | < 0.001 | 424 | -3.7 (-5.5, -2.0)^d^ | < 0.001 |
| Able to save money most weeks / you are comfortable | 940 | Reference |  | 883 | Reference |  |
| Area-level SES | 1,574 | Adj R^2^ = 0.036 |  | 1,531 | Adj R^2^ = 0.061 |  |
| Low | 327 | -6.9 (-8.7, -5.1) | < 0.001 | 315 | -5.2 (-7.1, -3.4)^e^ | < 0.001 |
| Medium | 596 | -3.7 (-5.2, -2.2) | < 0.001 | 580 | -3.0 (-4.5, -1.5)^e^ | < 0.001 |
| High | 651 | Reference |  | 636 | Reference |  |

n differs due to missing values for covariates.

^a^ Adjusted for age.

^b^ Adjusted for perceived social support.

^c^ Adjusted for age, education level, and latent classes of SLEs.

^d^ Adjusted for equivalised income, perceived social support, and latent classes of SLEs.

^e^ Adjusted for equivalised income.

Abbreviations: CI, confidence interval; SES, socioeconomic status; SLEs, stressful life events.
